# Inhibition of IGF1-R overcomes IGFBP7-induced chemotherapy resistance in T-ALL

**DOI:** 10.1186/s12885-015-1677-z

**Published:** 2015-10-08

**Authors:** Isabelle Bartram, Ulrike Erben, Jutta Ortiz-Tanchez, Katja Blunert, Cornelia Schlee, Martin Neumann, Sandra Heesch, Claudia D. Baldus

**Affiliations:** 1 Department of Hematology and Oncology, Campus Benjamin Franklin, Charité - Universitätsmedizin Berlin, Hindenburgdamm 30, Berlin, 12203 Germany; 2 Department of Gastroenterology, Infectiology and Rheumatology, Campus Benjamin Franklin, Charité - Universitätsmedizin Berlin, Hindenburgdamm 30, Berlin, 12203 Germany

**Keywords:** T-cell acute lymphoblastic leukemia, Chemotherapy resistance, Insulin-like growth factor-system, IGFBP7

## Abstract

**Background:**

T-cell acute lymphoblastic leukemia (T-ALL) is a genetically heterogeneous disease with the need for treatment optimization. Previously, high expression of Insulin-like growth factor binding protein 7 (IGFBP7), a member of the IGF system, was identified as negative prognostic factor in adult T-ALL patients. Since aberrant *IGFBP7* expression was observed in a variety of neoplasia and was relevant for prognosis in T-ALL, we investigated the functional role of *IGFBP7* in Jurkat and Molt-4 cells as *in vitro* models for T-ALL.

**Methods:**

Jurkat and Molt-4 cells were stably transfected with an IGFBP7 over-expression vector or the empty vector as control. Proliferation of the cells was assessed by WST-1 assays and cell cycle status was measured by flow-cytometry after BrDU/7-AAD staining. The effect of IGFBP7 over-expression on sensitivity to cytostatic drugs was determined in AnnexinV/7-AAD assays. IGF1-R protein expression was measured by Western Blot and flow-cytometric analysis. IGF1-R associated gene expression profiles were generated from microarray gene expression data of 86 T-ALL patients from the Microarrays Innovations in Leukemia (MILE) multicenter study.

**Results:**

IGFBP7*-*transfected Jurkat cells proliferated less, leading to a longer survival in a nutrient–limited environment. Both IGFBP7-transfected Jurkat and Molt-4 cells showed an arrest in the G0/G1 cell cycle phase. Furthermore, Jurkat IGFBP7-transfected cells were resistant to vincristine and asparaginase treatment. Surface expression and whole protein measurement of IGF1-R protein expression showed a reduced abundance of the receptor after IGFBP7 transfection in Jurkat cells. Interestingly, combination of the IGF1-R inhibitor NPV-AEW541 restored sensitivity to vincristine in IGFBP7-transfected cells. Additionally, IGF1-R associated GEP revealed an up-regulation of important drivers of T-ALL pathogenesis and regulators of chemo-resistance and apoptosis such as NOTCH1, BCL-2, PRKCI, and TP53.

**Conclusion:**

This study revealed a proliferation inhibiting effect of IGFBP7 by G0/G1 arrest and a drug resistance-inducing effect of IGFBP7 against vincristine and asparaginase in T-ALL. These results provide a model for the previously observed association between high *IGFBP7* expression and chemotherapy failure in T-ALL patients. Since the resistance against vincristine was abolished by IGF1-R inhibition, *IGFBP7* could serve as biomarker for patients who may benefit from therapies including IGF1-R inhibitors in combination with chemotherapy.

**Electronic supplementary material:**

The online version of this article (doi:10.1186/s12885-015-1677-z) contains supplementary material, which is available to authorized users.

## Background

Acute lymphoblastic leukemia (ALL) is a heterogeneous disease which survival rate has greatly improved during the last decades due to improvement in risk stratification and adapted treatment strategies. Despite these advances, adult T-cell acute lymphoblastic leukemia (T-ALL) patients only have a survival of 40–70 %, depending on protocol and age group. Thus the urgent need for the development of novel therapy approaches remains [1, 2].

Insulin-like growth factor binding protein 7 (IGFBP7) is a part of the IGFBP family (IGFBP1-7) of proteins that bind insulin-like growth factors (IGFs) to regulate their bioavailability and transport. IGFs in turn bind to IGF- receptor 1 (IGF1-R), which has a tyrosine-kinase domain and acts through the PI3K-AKT and RAS-RAF-MAPK pathways [3]. In addition, IGFBPs also have IGF-independent actions but the respective molecular mechanism of cellular transduction have only been described for some of them [4, 5]. For example IGFBP3 was revealed to bind to a receptor and nuclear localization was observed for IGFBP2, −3 and −5 [6–8].

IGFBP7 has been described as tumor suppressor in several human cancers. In breast, colorectal and hepatocellular carcinoma patients down-regulation of IGFBP7 in tumor cells was associated with a worse prognosis [9–11]. In hepatocellular carcinoma and malignant melanoma IGFBP7 was even suggested as therapeutic agent as it inhibited tumor cell viability and promoted apoptosis *in vivo* and *in vitro* [12–14].

In leukemia, *IGFBP7* was reported to be co-expressed with the negative prognostic factor *brain and acute leukemia, cytoplasmic* (*BAALC*) in T-ALL and acute myeloid leukemia (AML) patients [15]. High *IGFBP7* mRNA expression was found to be associated with primary therapy resistance and negative outcome in T-ALL patients [15]. Similarly to findings of studies with different malignant cell lines, addition of rIGFBP7 was able to decrease proliferation of leukemic cell lines *in vitro* [12, 15–19].

Several studies have revealed a link between IGFBP7 and acute leukemia: in childhood AML and ALL *IGFBP7* was found to be up-regulated and high expression was associated with lower survival in precursor B-cell Ph(−) ALL patients [20, 21]. Also ALL blasts were found to be protected from asparaginase through *IGFBP7* expression induced by co-culture of the blasts with bone marrow stroma cells (BMSC) [21].

However, further actions and downstream targets of IGFBP7 in acute leukemia remain yet to be investigated. Thus, in this study we explored IGFBP7’s functional role in T-ALL and uncovered mechanisms of its actions directing drug resistance. Our study revealed IGFBP7-induced resistance against chemotherapeutic drugs in T-ALL. Additionally our results underscore an interaction of IGFBP7 and the IGF1-R.

## Methods

### Cell lines and culture

The human cell lines K562 (chronic myeloid leukemia; ACC-10), Jurkat (T-ALL; ACC-282), Molt-4 (T-ALL; ACC-362) and KG-1a (erythroleukemia; ACC-421) were purchased from the DSMZ (Braunschweig, Germany). Cells were maintained in RPMI1640 medium containing 25 mM HEPES, 2 mM L-glutamine, 1 mM sodium pyruvate, 100 U/mL penicillin and 100 mg/mL streptomycin (all from Merck Millipore, Darmstadt, Germany). Medium was supplemented with 10 % fetal bovine serum (20 % for Molt-4 cells; Linaris, Bettingen, Germany). Cell cultures were routinely checked for mycoplasma contamination using PCR (Merck Millipore).

### Plasmid constructs and transfection

Human cDNA prepared from normal BMSCs served as template for a standard PCR (Life Technologies, Regensburg, Germany) to amplify the *IGFBP7* transcript variant1 (accession number: NCBI NM_001553.2) using the primers 5’-CACCCCGCCATGGAG-3’ and 5’-TATAGCTCGGCACCTTCACC-3’. The 857 bp PCR product was cloned into vector pcDNA3.1myc/HisC (Life Technologies, Regensburg, Germany) for eukaryotic over-expression (pIGFBP7). Jurkat and Molt-4 cells were transfected with pIGFBP7 or the empty vector for as control (pCntrl) using the Nucleofector device (Lonza, Basel, Switzerland) according to the manufacturer’s instructions. After 24 hours, transfected cells received neomycin (800 μg/mL; Merck Millipore, Darmstadt, Germany) for three weeks before *IGFBP7* over-expression was confirmed by qRT-PCR. Cells were cloned by limiting dilution into 96-well plates (Nunc, Glostrup, Denmark). Cultures were kept in the presence of 800 μg/mL neomycin for an additional 4 weeks. Before conductions of experiments, cells were thawed, cultured for two weeks in the presence of neomycin and stable *IGFBP7* over-expression reconfirmed by qRT-PCR.

### RNA extraction and quantitative real-time PCR

RNA was isolated with the RNeasy Kit (Qiagen, Hilden, Germany) and transcribed into cDNA using MMLV reverse transcriptase (Epicentre, Chicago, USA). Quantitative real-time PCR (qRT-PCR) for *IGFBP7* was performed as previously described in a Sybr Green (Invitrogen GmbH, Karlsruhe, Germany) PCR assay [22] using the primers *IGFBP7*-forward: 5’-CATCACCCAGGTCAGCAAG-3’ and *IGFBP7*-reverse: 5’-TCACAGCTCAAGTACACCTG-3’. To measure *IGF1R* mRNA expression primers *IGF1R*-forward 5’-ACGGGGCGATCTCAAAAGTT-3’ and *IGF1R*-reverse 5’-CTCTCCGGCCATCTGAATCA-3’ were used. Expression of the house keeping gene *glyceraldehyde-3-phosphate dehydrogenase* (*GAPDH*) was used as internal control. Relative Expression values were expressed as 2-^ΔCT^ using the comparative cycle threshold (ct) method [23].

### ELISA

IGFBP7 as secreted protein is likely to act through paracrine/autocrine pathways; therefore its concentration in the conditioned medium from IGFBP7 over-expressing versus control cell lines was measured with a “Super-X” IGFBP7-ELISA kit (Antigenix America Inc., NY, USA) along the manufacturer’s instructions. To harvest conditioned medium, transfected cells were seeded at 1×10^6^ cells/ml and grown for four days. The cells were then separated from the medium by centrifugation for 10 min at 2000 rpm at 4 °C and the medium was treated with cOmplete Mini protease inhibitor cocktail (Roche, Basel, Switzerland) and stored at −80 °C until analysis.

### WST-1 proliferation assays

To determine the effect of IGFBP7 over-expression on proliferation of leukemic cell lines, the pCntrl or pIGFBP7 transfected clones were seeded in triplicates in different 96-well plates for separate time points. 1×10^5^ cells/well in 100 μl were seeded and incubated at 37 °C, 5 % CO_2_ for one to four days. At the endpoint of the experiment, 10 μl WST-1 reagent (Roche, Basel, Switzerland), diluted 1:2 with regular medium, was added to each well. Plates were then incubated for two hours to allow for the reduction of WST-1 reagent to soluble formazan by the respiratory chain of the viable cells. Absorbance was measured with a Sunrise (Tecan, Männedorf, Switzerland) microplate absorbance reader at 450 nm with a reference wavelength of 620 nm.

### Apoptosis assays

Apoptosis of cells treated with drugs or starvation-induced apoptosis was measured by staining for AnnexinV-phycoerythrin (FITC) and 7-amino-actinomycin D (7-AAD) using an AnnexinV–FITC apoptosis detection kit (BD Pharmingen, Heidelberg, Germany). For chemosensitivity-assays cells were seeded at 1×10^6^ cells/ml in triplicates and treated with vincristine (1 ng/ml), etoposide (1 μg/ml), cytarabine (araC) (1 μg/mL) or asparginase (1 IU/ml) in combination with DMSO alone or 500 nM IGF1-R-inhibitor NVP-AEW541 (AEW451; Novartis AG, Basel, Switzerland) dissolved in DMSO. Drug concentrations used in the assays were determined in prior WST-1 assays of different concentrations. Cells were harvested after 24 h and analyzed by FACSCalibur (BD Pharmingen, Heidelberg, Germany).

### BrDU cell cycle assays

Cell cycle analysis was performed using a BrDU-FITC kit (BD Pharmingen, Heidelberg, Germany) as described in manufacturer’s protocol. Before staining and FACS analysis, cells were incubated with 10 μM BrDU for four hours.

### IGF1-R FACS staining

IGF1-R surface expression was measured by a flowcytometric assay. Cells were seeded at 1×10^6^ cells/ml in duplicates, cultured normally for four days and stained with anti-IGF-IR antibody, clone αIR3 (Merck Millipore, Darmstadt, Germany) which recognizes the ~130 kDa α and the ~90 kDa β subunits of the IGF1-R. For isotype controls an unspecific mouse IgG_1_ was used (Santa Cruz Biotechnology, Dallas, USA). For FACS analysis samples were stained with anti-mouse IgG Alexa®488 (New England Biolabs, Ipswich, USA) as secondary antibody. The median fluorescence intensity (MFI) of the samples was normalized to the MFI of the isotype controls before pooling values of independent experiments.

### Western blot

Cells were seeded at 1×10^6^ cells/ml, incubated for two days, and lysed in RIPA buffer [20 mM Tris–HCl, 150 mM NaCL, 1 mM NaCl, 1 mM Na_2_-EDTA, 1 mM EGTA, 1 % NP-40, 1 % sodium deoxycholate, 2.5 mM sodium pyrophosphate, 1 mM b-glycerophosphate, 1 mM Na_3_VO_4_ and cOmplete Mini protease inhibitor cocktail (Roche, Basel, Switzerland)]. Whole cell extracts of 5×10^5^ cells were diluted in SDS-loading buffer and denatured for 5 min at 95 °C. The samples were then separated by 12.5 % SDS-polyacrylamide gel electrophoresis and blotted onto a 0.2 μm PDV membrane (Bio-Rad Laboratories Inc., Hercules, USA). After blocking in TBST 3 % BSA, blots were incubated with IGF-IRβ antibody (C-20, Santa Cruz Biotechnology, Dallas, USA) following by anti-rabbit-HRP (Santa Cruz Biotechnology, Dallas, USA) and developed with an ECL system (Western Lightning Plus ECL; PerkinElmer, Waltham, USA). As loading control, blots were stripped and incubated again with an anti-beta-actin antibody-HRP (Abcam, Cambridge, UK).

### Gene expression profiles

*IGF1-R*-associated GEP of an independent set of 86 adult T-ALL samples were generated from raw data obtained from the Microarrays Innovations in Leukemia (MILE) multicenter study (HG-U133 Plus 2.0 and HG-U133 A+B; Affymetrix, Santa Clara, CA, USA) [24]. All patients had given written informed consent to participate according to the Declaration of Helsinki and the MILE study design was approved by the ethics committees of the participating institutions [24]. As we have used this already existent dataset and the experiments we performed ourselves only included established cell lines, no additional approval by an ethics committee was necessary. Information about the MILE study is provided in the cited article [24].

For analysis the samples were divided into a low *IGF1-R* expression group (*n* = 42) and a high *IGF1-R* group (*n* = 41) according to the expression level of IGF1-R (represented by the median of the two probe sets 203628_at, 203627_at). In order to obtain gene expression signatures that discriminated between high and low *IGF1-R* expressers, samples in the high IGF1-R expression group were compared to samples in the low expression group. Genes were considered to be differentially expressed if their expression showed at least a 1.5-fold change and a FDR < 0.05. The data analyses were carried out with Partek Genomic Suite 6.6 (Partek Incorporated, St. Louis). For further analysis DAVID Bioinformatics Database was utilized to functionally annotate the genes up- or down-regulated in *IGF1R*-high patients (Gene Ontology: GOTERM_BP_FAT).

### Statistics

The statistical difference between values of two independent groups was tested using the nonparametric Mann–Whitney U-test. For paired values the non-parametric Wilcoxon matched-pair test was used. A *P*-value ≤ 0.05 (two-sided) was considered to indicate a significant difference. Statistical analyses were performed with Prism 5 software (GraphPad Software Inc., San Diego, USA).

## Results

### *IGFBP7* over-expression sustains proliferation of Jurkat cells

**Fig. 1:**
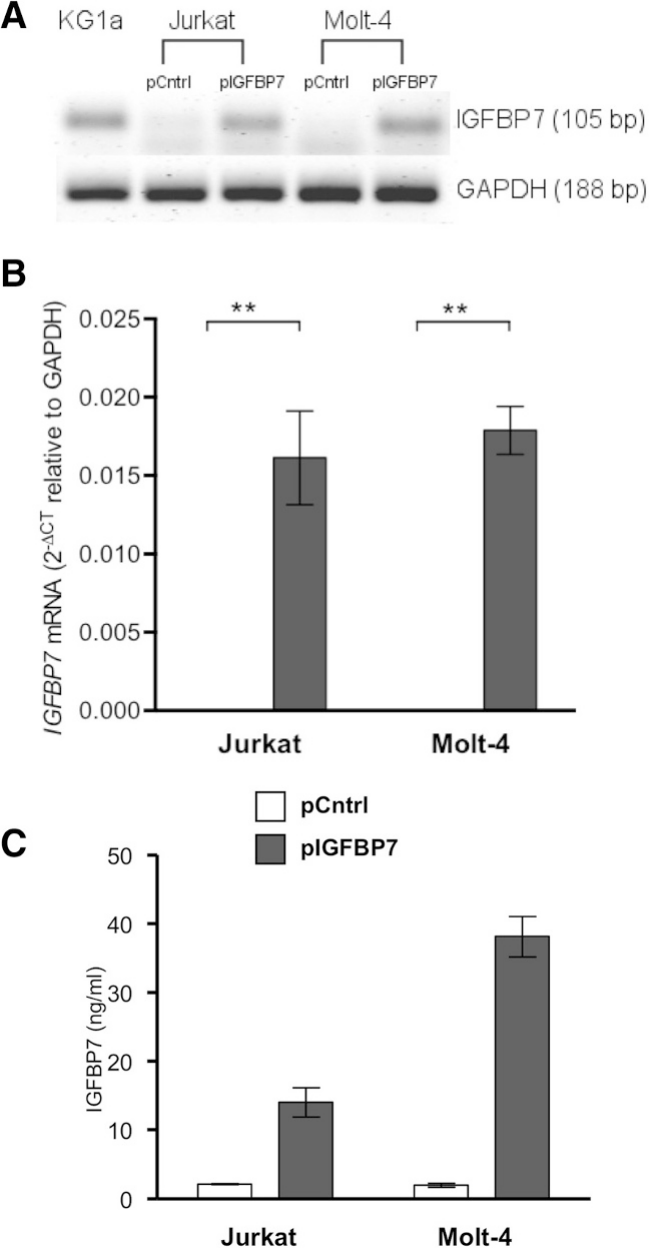
IGFBP7 expression in Jurkat and Molt-4 transfected for over-expression. Jurkat and Molt-4 cells were transfected with pCntrl or pIGFBP7 and cloned for 35 days in the presence of G418. **a**: *IGFBP7* or *GAPDH* mRNA levels were assessed after additional 24 h culture without G418 by RT-PCR. KG-1a served as control for constitutive *IGFBP7* mRNA expression. Images representative for four independent experiments show PCR products separated by agarose gel electrophoresis. **b**: *IGFBP7* mRNA expression was measured by Sybr Green qRT-PCR and normalized to *GAPDH* as housekeeping control. Mean values ± SEM of five duplicate determinations. **P-value ≤ 0.01 (Mann–Whitney-U test) **c**: Secreted IGFBP7 protein from Jurkat or Molt-4 clones was quantified from the supernatants by ELISA after four days of culture without G418. Mean values ± SEM of three triplicate determinations

In order to determine the effect of *IGFBP7* over-expression in TALL*, IGFBP7* was successfully over-expressed in Jurkat and in Molt-4 cells (mean mRNA level *IGFBP7*-expression increase: Jurkat 2643-fold, Molt-4 2273-fold; Fig. 1a and b; *P*-value = 0.008). Secreted IGFBP7 was detected in the conditioned medium of Jurkat and Molt-4 clones with a stably integrated *pcDNA3.1-IGFBP7* construct (referred to as pIGFBP7) (Fig. 1c). The secreted IGFBP7 protein concentration in the medium was increased 6.5-fold in Jurkat and 19.1-fold in Molt-4 cells as determined by ELISA.

**Fig. 2:**
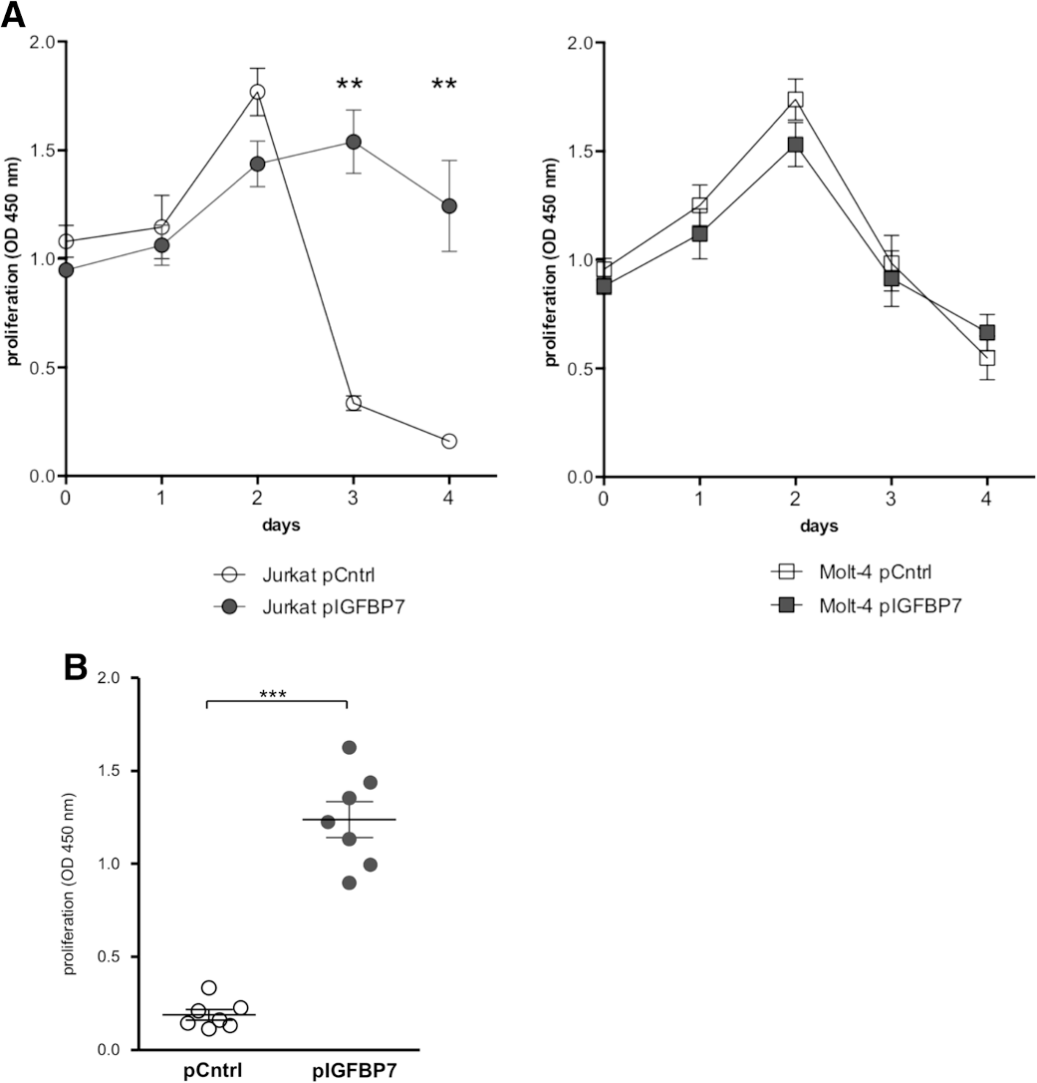
Proliferation of Jurkat clones over-expressing IGFBP7. Proliferation of Jurkat and Molt-4 clones transfected with pCntrl or pIGFBP7 was measured in a WST-1 assay. **a**: Five independent experiments over a time course of four days. Mean values ± SD of triplicate determinations. **P-value ≤ 0.01 (Mann–Whitney-U test) **b**: Proliferation at day four of culture of Jurkat cells. Mean values of 7 independent experiments done in triplicates are plotted; lines indicate mean values ± SEM. ***P-value ≤ 0.001 (Mann–Whitney-U test)

To assess the effect of IGFBP7 over-expression, pCntrl and pIGFBP7 transfected cells were grown for four days at a high cell concentration without medium exchange to promote starvation-induced apoptosis. The Jurkat pCntrl transfected cells initially showed a steep increase followed by a steep decrease of proliferation over the time course of four days. In contrast the pIGFBP7 transfected cells showed a more shallow increase of proliferation, which then remained at a constant level throughout the experiment (Fig. 2a). The difference of resistance against starvation-induced cell death was highly significant with pIGFBP7 transfected Jurkat cells showing 85 % more proliferation measured by WST-1 signal on day four compared to the pCntrl cells (*P*-value = 0.0006; Fig. 2b). The Molt-4 cells showed a somehow similar difference in the proliferation dynamics of pCntrl and pIGFBP7 transfected cells, but was not statistically significant (Fig. 2a).

### IGFBP7 over-expression induces G0/G1 arrest in T-ALL cells

**Fig. 3:**
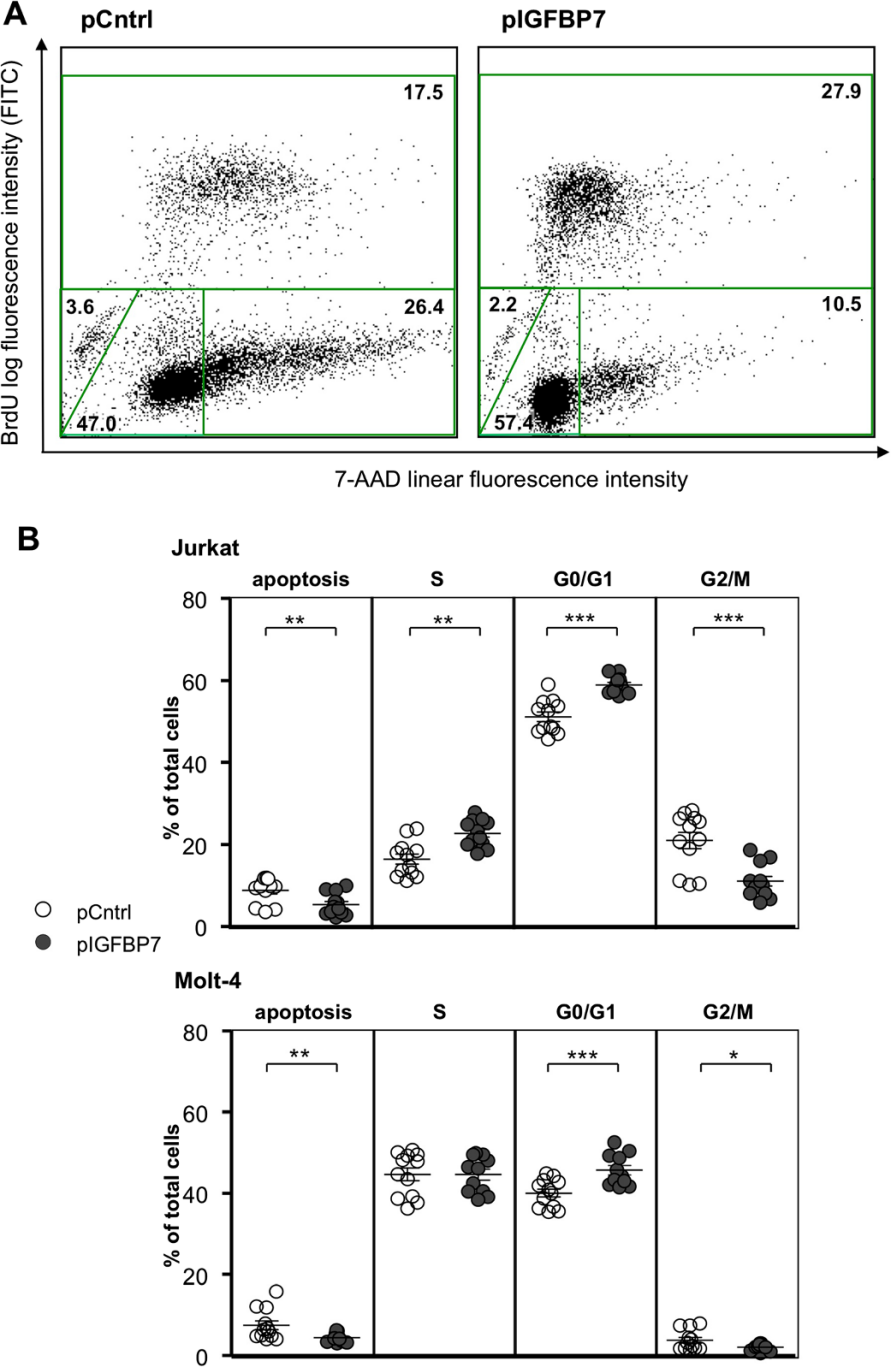
Cell cycle distribution of Jurkat and Molt-4 clones over-expressing IGFBP7. Clones transfected with pCntrl or pIGFBP7 were cultured for one (Molt-4) or three days (Jurkat). Cell cycle states were analyzed by flow cytometry after BrdU incorporation and subsequent antibody binding in combination with direct 7-AAD staining. **a**: Representative dot blots of transfected Jurkat cells show the gated populations: BrdU^+^ cells (upper region) were considered to be in S-phase; BrdU^−^7-AAD^high^ (lower right region) in G2/M, BrdU^−^7-AAD^low^ in G1/G0 (lower middle region) and BrdU^−^7-AAD^−^ apoptotic (lower left region). Percentages of cells within each region are indicated. **b**: Relative distribution of Jurkat and Molt-4 cells in cell cycle phases. Triplicates of four independent experiments are plotted; lines indicate mean values ± SEM; *P-value ≤ 0.05, **P-value ≤ 0.01, ***P-value ≤ 0.001 (Wilcoxon signed-rank test)

The effect of IGFBP7 over-expression on cell cycle dynamics in Jurkat and Molt-4 cells was measured with a BrDU assay. Both cell lines showed a significant change in cell cycle phases in pIGFBP7 compared to pCntrl cells (Fig. 3a and b). Jurkat pIGFBP7 cells had significantly more cells arrested in G0/G1 than pCntrl transfected cells (mean % of total cells: 59.0 vs. 51.2, *P*-value = 0.0005) and less in G2/M-phase (mean % of total cells: 11.1 vs. 21.0, *P*-value = 0.0005). IGFBP7-over-expressing Jurkat cells showed a higher percentage of cells in S-phase (mean % of total cells: 22.8 vs. 16.5, *P*-value = 0.005) and lower percentage of cells were apoptotic (mean % of total cells: 5.3 vs. 8.8, *P*-value = 0.002; Fig. 3a). Likewise, Molt-4 pIGFBP7 transfected cells were significantly more in G0/G1 arrest as compared to pCntrl transfected cells (mean % of total cells: 46.0 vs. 40.1, *P*-value = 0.001). *IGFBP7*-overexpressing Molt-4 cells were also slightly less in G2/M-phase (mean % of total cells: 2.1 vs. 3.8; *P*-value = 0.02) and less apoptotic (mean % of total cells: 4.4 vs. 7.5, *P*-value = 0.005) but did not show change in S-phase compared to controls, respectively.

### IGFBP7 induces resistance to chemotherapeutic drugs in Jurkat cells

**Fig. 4:**
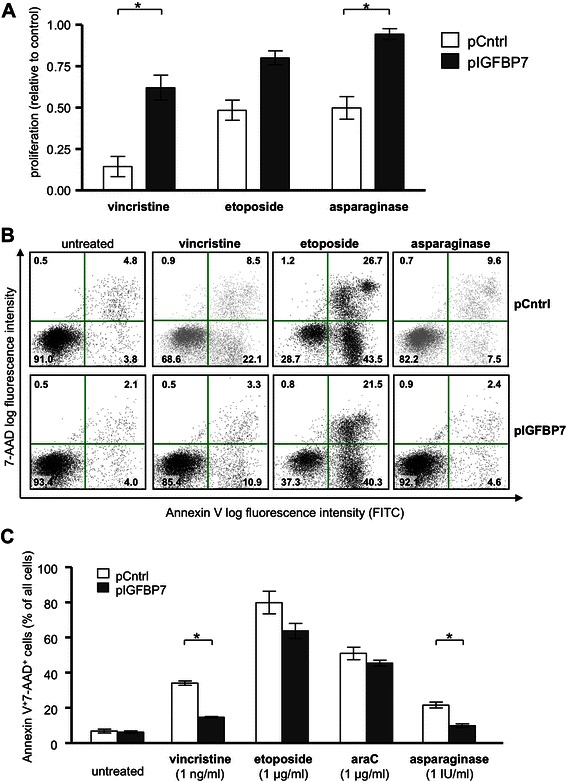
Cytostatic-drug treatment of a Jurkat clone overexpressing IGFBP7. Jurkat clones transfected with pCntrl or pIGFBP7 were incubated in the presence of vincristine, etoposide or asparaginase for 24 hours. **a**: A WST-1 assay was performed and proliferation upon cytostatic drug-treatment determined in relation to the respective untreated control (1). Mean values ± SEM of 4 independent experiments done in triplicates. *P-value ≤ 0.05 (Mann–Whitney-U-test) **b**: Cells stained with AnnexinV-FITC and 7-AAD were analyzed by flow cytometry. Representative dot plots out of four independent experiments. Percentages of cells within each quadrant are indicated. **c**: Early apoptotic (AnnexinV^+^7-AAD^−^) as well as late apoptotic and necrotic cells (AnnexinV^+^7-AAD^+^) were assessed as shown (**b**). Mean values ± SEM/SD of 4 independent experiments done in triplicates. *P-value ≤ 0.05 (Mann–Whitney-U test)

To test whether IGFBP7 over-expression influences the sensitivity of T-ALL cells to cytostatic drugs used in current ALL chemotherapy protocols, cells were incubated with vincristine, etoposide, or asparaginase for 24 h and proliferation was assessed. Jurkat pIGFBP7 transfected cells remained significantly more proliferative compared to pCntrl transfected cells when treated with vincristine (% of proliferation compared to untreated control: 65 vs. 22, *P*-value = 0.03) or asparaginase (% of proliferation compared to untreated control: 122 vs. 77, *P*-value = 0.03; Fig. 4a). No significant difference in proliferation between pCntrl and pIGFBP7 transfected Jurkat clones was measured after treatment with etoposide.

When cells in the same experimental setup were stained for early and late apoptosis markers AnnexinV and 7-AAD, a similar effect was measured: while 34.1 % pCntrl transfected cells were on average apoptotic (double positive for AnnexinV/7-ADD) after treated with vincristine, only 14.6 % of IGFBP7-overexpressing cells stained positive for AnnexinV/7-ADD (*n* = 4, *P* = 0.03; Fig. 4c). Treatment with asparaginase induced apoptosis in 21.6 % of pCntrl transfected cells and only 9.9 % of pIGFBP7 Jurkat cells (*n* = 4, *P*-value = 0.03). No significant effects of IGFBP7-overexpression on apoptosis were observed after treatment with etoposide or cytarabine.

### IGFBP7 over-expression induces reduction of IGF1-R protein in Jurkat cells

**Fig. 5:**
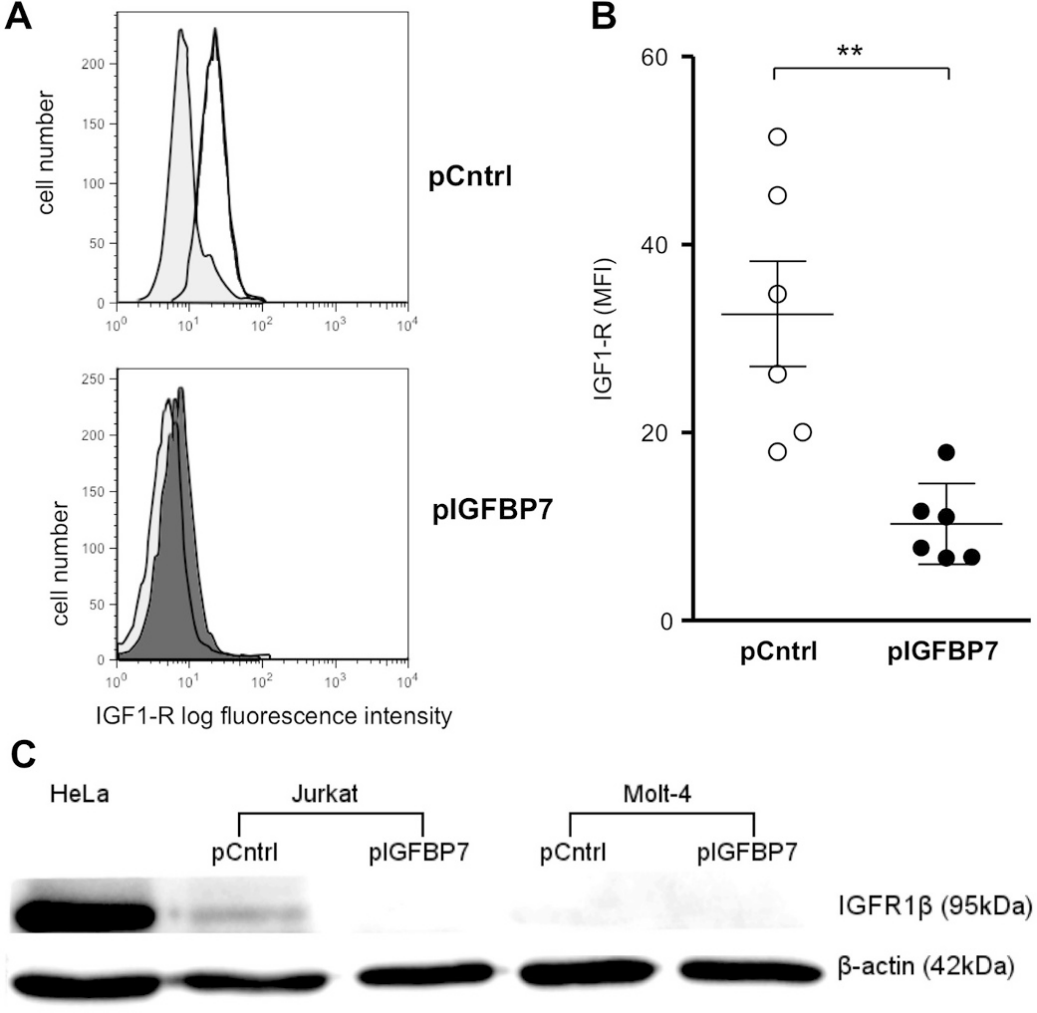
IGF1-R protein in Jurkat and Molt-4 clones over-expressing IGFBP7. **a**: Jurkat clones transfected with pCntrl or pIGFBP7 were incubated for four days without medium exchange. Cells were analyzed by flow cytometry for IGF1-R in viable cells according to their forward and side scatter properties. Representative dot plots show each clone with a respective isotype-matched negative control (light grey) from an exemplary out of five experiments. **b**: Results from five independent IGF-1R median fluorescence intensity (MFI) determinations in duplicates, lines indicate means and SEM. **P-value ≤ 0.01 (Mann–Whitney-U test) **c**: Jurkat or Molt-4 clones were cultured for one day and lysates subjected to Western-blot analysis for IGF1-Rβ and β-actin. The cell line HeLa served as positive control. The Image is representative for three independent experiments

IGF1-R surface expression was measured by flow cytometric staining in pIGFBP7 compared to pCntrl transfected Jurkat and Molt-4 clones. IGFBP7 over-expressing Jurkat cells showed a significantly lower level of IGF1R surface expression compared to control cells (Fig. 5a and b; 68.4 % less compared to control, *P*-value = 0.002). Molt-4 cells showed only a very low level of IGF1-R expression, which was not influenced by IGFBP7 over-expression (not shown). When whole cell lysates were analyzed for IGF1-R expression by western blot, in Jurkat pCntrl transfected IGF1-R was detected at 95 kDa, while Jurkat pIGFBP7 transfected cells did not express a detectable amount of IGF1R, underscoring the flow cytometric results. Molt-4 cells also showed no detectable protein expression of IGF1-R (Fig. 5c). When *IGF1-R* mRNA expression was determined by qRT-PCR, no significant difference was found for pCntrl or pIGFBP7 transfected cells (not shown). Thus, IGFBP7 over-expression resulted in post-transcriptional reduction of IGF1-R protein abundance in Jurkat cells.

### IGFBP7-induced resistance is overcome by IGF1R inhibition in Jurkat cells

Since IGFBP7 over-expression modulated IGF1-R protein abundance, we combined the treatment of chemotherapeutic drugs with an IGF1-R inhibitor to test whether the IGFBP7-induced chemotherapy resistance could be modulated by combination with an IGF1-R inhibitor. Jurkat pIGFBP7 transfected cells were more sensitive to the exposure of the IGF1-R inhibitor AEW541 than control clones (mean % of apoptotic cells: 64.2 vs. 41.5, *P*-value = 0.03). Combination of vincristine with AEW541 reversed the resistance-inducing effect of IGFBP7: Jurkat cells over expressing IGFBP7 regained sensitivity to vincristine with 79.8 % of pIGFBP7 transfected being apoptotic when treated in combination with AEW541 compared to only 14.6 % when treated with vincristine alone (*P*-value = 0.03). pCntrl-transfected Jurkat cells were less sensitive compared to pIGFBP7-transfected cells to the combination treatment (mean % of apoptotic cells: 51.9 vs. 79.8 %; *P*-value = 0.03).

**Fig. 6:**
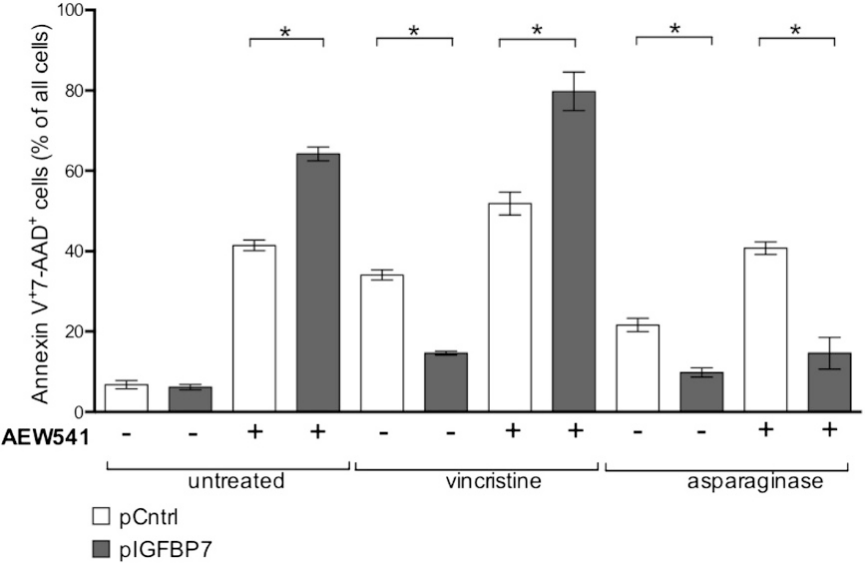
Apoptosis in Jurkat clone over-expressing IGFBP7 upon treatment with cytostatic drugs in combination with IGF1-R inhibition. Jurkat clones transfected with pCntrl or pIGFBP7 were incubated for one day with different drugs in combination with 500 nM IGF1-R-inhibitor AEW541. Cells stained with AnnexinV and 7‑AAD were assessed by flow cytometry. Mean values ± SEM of 4 independent experiments done in triplicates; *P-value≤ 0.05 (Mann-Whitney-U test)

In contrast, pIGFBP7 transfected Jurkat cells remained significantly less sensitive to asparaginase when treated in combination with AEW541 (mean % of apoptotic cells: 14.5 vs. 40.8, *P*-value = 0.03; Fig. 6).

### *IGF1-R* associated gene expression profiles of T-ALL patients

To further explore the relevance of IGF1-R signaling in primary T-ALL samples, we analyzed gene expression data from 86 T-ALL patients for *IGF1-R* co-regulated genes. In patient samples highly expressing *IGF1-R* 257 probe sets were differentially expressed. They corresponded to 226 unique genes, hypothetical genes, proteins and open reading frames. Of those 165 genes were up-regulated and 61 down-regulated. For a complete list of differentially expressed probe sets see Additional file 1: Table S1 and Table S2.

In Gene Ontology analyses, genes highly expressed and co-regulated with *IGF1-R* were significantly enriched in categories related to apoptosis (“anti-apoptosis”, *P*-value = 0.01; “negative regulation of cell death”, *P*-value = 0.01; “negative regulation of programmed cell death”, *P*-value = 0.01). The differentially expressed genes in these categories included the anti-apoptotic gene *BCL2*, genes related to chemo-resistance like *ANXA4* and *PRKCI,* as well as pro-apoptotic tumor suppressor genes *TP53* and *DAPK1* [25–29]. Interestingly, also genes involved in leukemogenesis like *NOTCH1* and *HELLS,* suggesting a role of IGF1-R signaling not only in maintenance, but also in initial transformation of leukemic blasts (Table 1) [30, 31].

**Table 1:** Probe sets over-expressed in high *IGF1-R* group which cluster in apoptosis-related Gene Ontology categories

GOTERM_BP_FAT category		*P*-value	Fold-enrichment	Gene symbols
GO:0006916	Anti-apoptosis	0.01	3.83	ANXA4, STAMBP, DAPK1, BCL2, PRKCI, HELLS
GO:0043069	Negative regulation of programmed cell death	0.01	2.83	ANXA4, TP53, STAMBP, DAPK1, BCL2, PRKCI, NOTCH1, HELLS
GO:0060548	Negative regulation of cell death	0.01	2.82	ANXA4, TP53, STAMBP, DAPK1, BCL2, PRKCI, NOTCH1, HELLS

Differentially down-regulated genes in *IGF1-R*-high T-ALL patients were enriched in the categories induction of apoptosis (*P*-value = 0.02) and induction of programmed cell death (*P*-value = 0.02), the most functionally established being pro-apoptotic gene *BCL2L11* (Table 2).

**Table 2:** Probe sets under-expressed in high *IGF1-R* group which cluster in apoptosis-related Gene Ontology categories

GOTERM_BP_FAT category		*P*-value	Fold-enrichment	Gene symbols
GO:0006917	Induction of apoptosis	0.02	4.60	DDX20, BCL2L11, MAPK8, TNFSF8, ARHGEF7
GO:0006917	Induction of programmed cell death	0.02	4.58	DDX20, BCL2L11, MAPK8, TNFSF8, ARHGEF7

## Discussion

High expression of *IGFBP7* was found to be associated with poor survival and predicted primary therapy resistance in T-ALL patients, while treatment of leukemic cells lines with recombinant protein reduced proliferation [15]. Here we investigated the functional role of IGFBP7 in acute leukemia and its possible mode of action.

In the T-ALL cell line Jurkat, pIGFBP7 transfection resulted in a prolonged viability of cells in a starvation environment. Cell cycle analysis displayed a significant change in the cell cycle distribution in response to IGFBP7 over-expression in both Jurkat and Molt-4 cells: significantly more cells were in G0/G1-phase and less apoptotic or in G2/M-phase. In Jurkat cells also more IGFBP7-overexpressing cells were in S-phase.

Extended G0/G1 arrest is one way of evading toxicity of cytostatic drugs, since their mode of action is by disturbing cell division [32]. When treated with vincristine, a mitotic inhibitor, which binds to microtubulins during metaphase, a significantly reduced proliferation and apoptosis rate was seen for pIGFBP7 transfected Jurkat cells. A previous study had found that IGFBP7 increased asparagine synthase expression in ALL cells [21], possibly explaining the resistance of IGFBP7-overexpressing Jurkat cells against asparaginase. Also for etoposide, a slight but not significant resistance effect was seen, for cytarabine it was even smaller. Since vincristine was the only tested drug that is exclusively active in metaphase, these results point to a cell cycle phase specificity of IGFBP7-induced drug resistance. Since vincristine and asparaginase are both routinely used in current chemotherapy regimens the results also provide an explanation for the observed link between aberrantly high *IGFBP7* expression and chemotherapy resistance in T-ALL patients.

A previous study had shown that IGFBP7 binds to the extracellular domain of the IGF1-R [33]. In our setting IGFBP7-overexpression resulted not only in a lower surface abundance of the receptor but also lower total IGF1-R protein expression. Since *IGF1-R* mRNA expression levels were not affected, IGFBP7 likely has a post-transcriptional effect of on IGF1-R protein abundance.

When IGFBP7 over-expressing Jurkat cells were treated with the IGF1-R inhibitor AEW541 in combination with cytostatic drugs, the IGFBP7-induced resistance to vincristine was overcome, but not the resistance against asparaginase. Since the latter is not acting through cell cycle inhibition, but through hydrolysis of asparagine, the results further underscore the mode of action of IGFBP7-induced resistance by G0/G1 arrest through interaction with IGF1-R.

Interestingly Molt-4, another T-ALL cell line, did not show significant changes in proliferation in a starvation environment and no significant resistance to cytostatic drugs with respect to IGFBP7 over expression (data not included). Cell cycle changes were also observed between pCntrl and pIGFBP7 transfected Molt-4 cells, but to a lesser extent. Molt-4 cells natively express IGF1-R at a very low level, which might account for the observed reduced impact of IGFBP7 over-expression in Molt-4 compared to Jurkat cells.

Intriguingly, a recent study on AML cell lines showed different results to our findings in T-ALL [34]. Here, several AML cell lines were less viable and more apoptotic in serum-reduced medium after *IGFBP7* over expression. The authors also showed a block at G2-phase and a decrease in G0/G1-phase. Moreover the study found no reduction of IGF1-R expression when IGFBP7 was over-expressed and most remarkably the utilized AML cell lines were not resistant but sensitized for the induction of cell death by chemotherapeutic drugs when rIGFBP7 was added. This difference to our results in T-ALL cell lines is likely due to highly tissue and disease specific actions of IGFBP7, which will need to be further investigated. It is also strikingly that the aforementioned study showed a better prognosis of *IGFBP7* high expressing AML patients, while in our ALL study cohort no such associated had been observed [15].

IGF1-R inhibitors are a potential therapeutic option in leukemia patients since *in vitro* IGF1-R inhibition reduced proliferation of leukemic cells [35–37]. So far, clinical trials with various solid tumor patients unfortunately showed only a few responders to small molecules or antibodies targeting the IGF1-R [38]. In acute leukemia patients, no studies with reagents exclusively targeting IGF1-R have been conducted. However, our analyses underscore the potential importance of IGF1-R in T-ALL. The *IGF1-R*-associated GEP further underline the biological relevance of IGF1-R in T-ALL pathogenesis as differentially up- and down-regulated genes were enriched for several known players in leukemia such as NOTCH1. Aberrant NOTCH1 signaling is a potent driver of malignant T-cell transformation and it was previously shown to up-regulate *IGF1-R* in T-ALL cells [39, 40]. Likewise, BCL-2 plays an important role in chemo-resistance and pro-survival signaling and was shown to be regulated by the IGF signaling cascade [41–43].

## Conclusion

In summary, our results show that a combination of chemotherapy with IGF1-R inhibition could improve elimination of leukemic blasts in patients who are possibly more prone to chemotherapy resistance due to high IGFBP7 expression and at the same time express IGF1-R at a substantial level. It has been noted before that IGF1-R inhibitor trials possibly lack the correct predictive biomarkers to stratify patients that are more likely to benefit of molecular directed therapies [38, 44]. IGFBP7, in combination with IGF1-R expression, could serve as such a marker.

## Additional file

Additional file 1: Table S1. Probe sets in the *IGF1-R* signatures that are over-expressed in the high *IGF1-R* group. **Table S2.** Probe sets in the *IGF1-R* signatures that are under-expressed in the high *IGF1-R* group. (PDF 209 kb)
