# Supplementary material for: Inhibition of IGF1-R overcomes IGFBP7-induced chemotherapy resistance in T-ALL
Source: BMC Cancer. 2015 Oct 8;15:663. doi: 10.1186/s12885-015-1677-z (PMC4599323; doi:10.1186/s12885-015-1677-z)
Supplement: Additional file 1: Table S1. — Probe sets in the IGF1-R signatures that are over-expressed in the high IGF1-R group. Table S2. Probe sets in the IGF1-R signatures that are under-expressed in the high IGF1-R group. (PDF 209 kb) [file 12885_2015_1677_MOESM1_ESM.pdf]

Table S1. Probe sets in the *IGF1-R* signatures that are over-expressed in the high *IGF1-R* group

Probeset ID	Gene Symbol	Gene Title	Fold-Change (high vs. low)	P-value
AFFX-HUMRGE/M10098_5_at	LINC00273	long intergenic non-protein coding RNA 273	4,53	0,000
AFFX-HUMRGE/M10098_M_at	---	---	2,80	0,000
203628_at	IGF1R	insulin-like growth factor 1 receptor	2,38	0,000
218847_at	IGF2BP2	insulin-like growth factor 2 mRNA binding protein 2	2,35	0,001
AFFX-HUMRGE/M10098_3_at	---	---	2,29	0,001
203685_at	BCL2	B-cell CLL/lymphoma 2	2,25	0,001
203895_at	PLCB4	phospholipase C, beta 4	2,19	0,003
204114_at	NID2	nidogen 2 (osteonidogen)	2,19	0,000
AFFX-M27830_5_at	---	---	2,17	0,002
241833_at	PEX5L	peroxisomal biogenesis factor 5-like	2,07	0,005
213998_s_at	DDX1	DEAD (Asp-Glu-Ala-Asp) box helicase 17	2,05	0,000
201753_s_at	ADD3	adducin 3 (gamma)	2,02	0,000
218055_s_at	WDR41	WD repeat domain 41	1,99	0,001
203627_at	IGF1R	insulin-like growth factor 1 receptor	1,97	0,000
203440_at	CDH2	cadherin 2, type 1, N-cadherin (neuronal)	1,97	0,001
203148_s_at	TRIM14	tripartite motif containing 14	1,93	0,001
203896_s_at	PLCB4	phospholipase C, beta 4	1,93	0,001
220952_s_at	PLEKHA5	pleckstrin homology domain containing, family A member 5	1,92	0,001
218397_at	FANCL	Fanconi anemia, complementation group L	1,91	0,000
225330_at	IGF1R	insulin-like growth factor 1 receptor	1,91	0,000
220220_at	LRRC37A4P	leucine rich repeat containing 37, member A4, pseudogene	1,90	0,000
215679_at	Hs.32769	MRNA full length insert cDNA clone EUROIMAGE 362430	1,90	0,000
211795_s_at	FYB	FYN binding protein	1,90	0,005
217552_x_at	CR1	complement component (3b/4b) receptor 1 (Knops blood group)	1,89	0,002
219017_at	ETNK1	ethanolamine kinase 1	1,88	0,000
206492_at	FHIT	fragile histidine triad	1,88	0,001
213478_at	KAZN	kazrin, periplakin interacting protein	1,87	0,002
205882_x_at	ADD3	adducin 3 (gamma)	1,87	0,000
202502_at	ACADM	acyl-CoA dehydrogenase, C-4 to C-12 straight chain	1,86	0,001
201393_s_at	IGF2R	insulin-like growth factor 2 receptor	1,84	0,000
201392_s_at	IGF2R	insulin-like growth factor 2 receptor	1,84	0,000
201034_at	ADD3	adducin 3 (gamma)	1,82	0,004
201503_at	G3BP1	GTPase activating protein (SH3 domain) binding protein 1	1,81	0,001
212607_at	AKT3	v-akt murine thymoma viral oncogene homolog 3 (protein kinase B, gamma)	1,79	0,000
206232_s_at	B4GALT6	UDP-Gal:betaGlcNAc beta 1,4-galactosyltransferase, polypeptide 6	1,79	0,000
215143_at	DPY19L2P2	dpy-19-like 2 pseudogene 2 (C. elegans)	1,79	0,001
38671_at	PLXND1	plexin D1	1,78	0,002

220085_at	HELLS	helicase, lymphoid-specific	1,78	0,002
204226_at	STAU2	staufen double-stranded RNA binding protein 2	1,77	0,000
201661_s_at	ACSL3	acyl-CoA synthetase long-chain family member 3	1,76	0,000
213939_s_at	RUFY3	RUN and FYVE domain containing 3	1,75	0,001
219806_s_at	SMCO4	single-pass membrane protein with coiled-coil domains 4	1,74	0,000
201752_s_at	ADD3	adducin 3 (gamma)	1,74	0,000
205077_s_at	PIGF	phosphatidylinositol glycan anchor biosynthesis, class F	1,74	0,000
226085_at	CBX5	chromobox homolog 5	1,74	0,001
213888_s_at	TRAF3IP3	TRAF3 interacting protein 3	1,73	0,001
201301_s_at	ANXA4	annexin A4	1,73	0,005
203401_at	PRPS2	phosphoribosyl pyrophosphate synthetase 2	1,72	0,002
203491_s_at	CEP57	centrosomal protein 57kDa	1,72	0,000
206828_at	TXK	TXK tyrosine kinase	1,72	0,001
215894_at	PTGDR	prostaglandin D2 receptor (DP)	1,71	0,007
203282_at	GBE1	glucan (1,4-alpha-), branching enzyme 1	1,70	0,000
218303_x_at	KRCC1	lysine-rich coiled-coil 1	1,70	0,001
202451_at	GTF2H1	general transcription factor IIH, polypeptide 1, 62kDa	1,70	0,003
219055_at	SRBD1	S1 RNA binding domain 1	1,70	0,001
206233_at	B4GALT6	UDP-Gal:betaGlcNAc beta 1,4-galactosyltransferase, polypeptide 6	1,69	0,002
213446_s_at	IQGAP1	IQ motif containing GTPase activating protein 1	1,69	0,000
204049_s_at	PHACTR2	phosphatase and actin regulator 2	1,68	0,001
203343_at	UGDH	UDP-glucose 6-dehydrogenase	1,67	0,000
203243_s_at	PDLIM5	PDZ and LIM domain 5	1,67	0,002
218984_at	PUS7	pseudouridylate synthase 7 homolog (S. cerevisiae)	1,67	0,001
203139_at	DAPK1	death-associated protein kinase 1	1,66	0,007
221527_s_at	PARD3	par-3 partitioning defective 3 homolog (C. elegans)	1,66	0,002
213698_at	ZMYM6 /// ZMYM6NB	zinc finger, MYM-type 6 /// ZMYM6 neighbor	1,66	0,000
209982_s_at	NRXN2	neurexin 2	1,66	0,000
208875_s_at	PAK2	p21 protein (Cdc42/Rac)-activated kinase 2	1,66	0,001
213742_at	SRSF11	serine/arginine-rich splicing factor 11	1,66	0,000
207057_at	SLC16A7	solute carrier family 16, member 7 (monocarboxylic acid transporter 2)	1,66	0,000
207143_at	CDK6	cyclin-dependent kinase 6	1,66	0,000
209829_at	FAM65B	family with sequence similarity 65, member B	1,66	0,000
206918_s_at	CPNE1	copine I	1,66	0,000
205471_s_at	DACH1	dachshund homolog 1 (Drosophila)	1,65	0,000
218197_s_at	OXR1	oxidation resistance 1	1,65	0,000
209285_s_at	FAM208A	family with sequence similarity 208, member A	1,65	0,000
201013_s_at	PAICS	phosphoribosylaminoimidazole carboxylase, phosphoribosylaminoimidazole succinocarboxami	1,65	0,002
203182_s_at	SRPK2	SRSF protein kinase 2	1,65	0,005
213331_s_at	NEK1	NIMA-related kinase 1	1,64	0,000
213677_s_at	PMS1	PMS1 postmeiotic segregation increased 1 (S. cerevisiae)	1,64	0,003
207564_x_at	OGT	O-linked N-acetylglucosamine (GlcNAc) transferase	1,63	0,000

209049_s_at	ZMYND8	zinc finger, MYND-type containing 8	1,62	0,000
204236_at	FLI1	Friend leukemia virus integration 1	1,62	0,003
218138_at	MKKS	McKusick-Kaufman syndrome	1,62	0,003
203791_at	DMXL1	Dmx-like 1	1,62	0,000
213156_at	MIR568	MicroRNA 568	1,62	0,001
213574_s_at	KPNB1	karyopherin (importin) beta 1	1,61	0,001
215789_s_at	AJAP1	adherens junctions associated protein 1	1,61	0,004
211360_s_at	ITPR2	inositol 1,4,5-trisphosphate receptor, type 2	1,61	0,000
201813_s_at	TBC1D5	TBC1 domain family, member 5	1,61	0,000
212653_s_at	EHBP1	EH domain binding protein 1	1,61	0,001
209754_s_at	TMPO	thymopoietin	1,61	0,001
218902_at	NOTCH1	notch 1	1,61	0,003
219008_at	C2orf43	chromosome 2 open reading frame 43	1,61	0,001
211794_at	FYB	FYN binding protein	1,60	0,000
213229_at	DICER1	dicer 1, ribonuclease type III	1,60	0,000
201309_x_at	NREP	neuronal regeneration related protein	1,60	0,004
212609_s_at	AKT3	v-akt murine thymoma viral oncogene homolog 3 (protein kinase B, gamma)	1,60	0,000
201801_s_at	SLC29A1	solute carrier family 29 (nucleoside transporters), member 1	1,60	0,000
210251_s_at	RUFY3	RUN and FYVE domain containing 3	1,60	0,002
215726_s_at	CYB5A	cytochrome b5 type A (microsomal)	1,60	0,003
220429_at	NDST3	N-deacetylase/N-sulfotransferase (heparan glucosaminyl) 3	1,59	0,004
219938_s_at	PSTPIP2	proline-serine-threonine phosphatase interacting protein 2	1,59	0,006
210653_s_at	BCKDHB	branched chain keto acid dehydrogenase E1, beta polypeptide	1,59	0,001
219264_s_at	PPP2R3B	protein phosphatase 2, regulatory subunit B", beta	1,59	0,001
221589_s_at	ALDH6A1	aldehyde dehydrogenase 6 family, member A1	1,59	0,004
212175_s_at	AK2	adenylate kinase 2	1,59	0,002
203077_s_at	SMAD2	SMAD family member 2	1,59	0,000
220235_s_at	LRIF1	ligand dependent nuclear receptor interacting factor 1	1,59	0,003
213188_s_at	MINA	MYC induced nuclear antigen	1,58	0,002
203067_at	PDHX	pyruvate dehydrogenase complex, component X	1,58	0,000
218545_at	CCDC91	coiled-coil domain containing 91	1,58	0,002
218130_at	C17orf62	chromosome 17 open reading frame 62	1,58	0,000
211052_s_at	TBCD	tubulin folding cofactor D	1,57	0,003
213593_s_at	TRA2A	transformer 2 alpha homolog (Drosophila)	1,57	0,002
217484_at	CR1	complement component (3b/4b) receptor 1 (Knops blood group)	1,57	0,002
217869_at	HSD17B12	hydroxysteroid (17-beta) dehydrogenase 12	1,57	0,001
218209_s_at	RPRD1A	regulation of nuclear pre-mRNA domain containing 1A	1,57	0,000
204354_at	POT1	protection of telomeres 1	1,57	0,001
205871_at	PLGLA /// PLGLB1 /// PLGLB2	plasminogen-like A (pseudogene) /// plasminogen-like B1 /// plasminogen-like B2	1,57	0,000
219426_at	AGO3	argonaute RISC catalytic component 3	1,57	0,000
203147_s_at	TRIM14	tripartite motif containing 14	1,57	0,000
221058_s_at	CKLF	chemokine-like factor	1,57	0,000
202742_s_at	PRKACB	protein kinase, cAMP-dependent, catalytic, beta	1,57	0,005
217894_at	KCTD3	potassium channel tetramerisation domain	1,56	0,001

		containing 3		
212115_at	HN1L	hematological and neurological expressed 1-like	1,56	0,003
208877_at	PAK2	p21 protein (Cdc42/Rac)-activated kinase 2	1,56	0,000
204839_at	POP5	processing of precursor 5, ribonuclease P/MRP subunit (S. cerevisiae)	1,56	0,005
206518_s_at	RGS9	regulator of G-protein signaling 9	1,56	0,002
57739_at	DND1	dead end homolog 1 (zebrafish)	1,56	0,001
213573_at	KPNB1	karyopherin (importin) beta 1	1,56	0,003
216028_at	Hs.596944	Full length insert cDNA clone YY74A07	1,56	0,000
208953_at	LARP4B	La ribonucleoprotein domain family, member 4B	1,56	0,000
204019_s_at	SH3YL1	SH3 domain containing, Ysc84-like 1 (S. cerevisiae)	1,56	0,001
205945_at	IL6R	interleukin 6 receptor	1,55	0,005
201746_at	TP53	tumor protein p53	1,55	0,004
200967_at	PPIB	peptidylprolyl isomerase B (cyclophilin B)	1,55	0,006
202661_at	ITPR2	inositol 1,4,5-trisphosphate receptor, type 2	1,55	0,001
204068_at	STK3	serine/threonine kinase 3	1,55	0,001
212307_s_at	OGT	O-linked N-acetylglucosamine (GlcNAc) transferase	1,55	0,000
218135_at	ERGIC2	ERGIC and golgi 2	1,55	0,001
220295_x_at	DEPDC1	DEP domain containing 1	1,55	0,002
203080_s_at	BAZ2B	bromodomain adjacent to zinc finger domain, 2B	1,55	0,002
217858_s_at	ARMCX3	armadillo repeat containing, X-linked 3	1,55	0,001
204521_at	FAM216A	family with sequence similarity 216, member A	1,55	0,004
213302_at	PFAS	phosphoribosylformylglycinamidine synthase	1,55	0,001
219644_at	CCDC41	coiled-coil domain containing 41	1,55	0,000
213391_at	DPY19L4	dpy-19-like 4 (C. elegans)	1,55	0,000
213189_at	MINA	MYC induced nuclear antigen	1,54	0,001
203275_at	IRF2	interferon regulatory factor 2	1,54	0,003
202631_s_at	APPBP2	amyloid beta precursor protein (cytoplasmic tail) binding protein 2	1,54	0,000
213170_at	GPX7	glutathione peroxidase 7	1,54	0,000
221208_s_at	MSANTD2	Myb/SANT-like DNA-binding domain containing 2	1,54	0,000
209943_at	FBXL4	F-box and leucine-rich repeat protein 4	1,54	0,000
200885_at	RHOC	ras homolog family member C	1,54	0,002
213140_s_at	SS18L1	synovial sarcoma translocation gene on chromosome 18-like 1	1,53	0,000
209678_s_at	PRKCI	protein kinase C, iota	1,53	0,000
204634_at	NEK4	NIMA-related kinase 4	1,53	0,006
213887_s_at	POLR2E	polymerase (RNA) II (DNA directed) polypeptide E, 25kDa	1,53	0,002
212174_at	AK2	adenylate kinase 2	1,53	0,002
221829_s_at	TNPO1	transportin 1	1,53	0,002
220122_at	MCTP1	multiple C2 domains, transmembrane 1	1,53	0,002
222376_at	ENSG00000248161		1,53	0,000
209084_s_at	RAB28	RAB28, member RAS oncogene family	1,53	0,000
217627_at	ZNF573	zinc finger protein 573	1,52	0,001
201737_s_at	MARCH06	membrane-associated ring finger (C3HC4) 6, E3 ubiquitin protein ligase	1,52	0,000
202811_at	STAMBP	STAM binding protein	1,52	0,003
36553_at	ASMTL	acetylserotonin O-methyltransferase-like	1,52	0,000

218967_s_at	PTER	phosphotriesterase related	1,52	0,003
203257_s_at	C11orf49	chromosome 11 open reading frame 49	1,52	0,001
209817_at	PPP3CB	protein phosphatase 3, catalytic subunit, beta isozyme	1,52	0,000
210114_at	INVS	inversin	1,52	0,000
203075_at	SMAD2	SMAD family member 2	1,52	0,001
219109_at	SPAG16	sperm associated antigen 16	1,52	0,002
201759_at	TBCD	tubulin folding cofactor D	1,52	0,000
214773_x_at	TIPRL	TIP41, TOR signaling pathway regulator-like (S. cerevisiae)	1,51	0,000
202660_at	ITPR2	inositol 1,4,5-trisphosphate receptor, type 2	1,51	0,007
204917_s_at	MLLT3	myeloid/lymphoid or mixed-lineage leukemia (trithorax homolog, Drosophila); translocate	1,51	0,003
204091_at	PDE6D	phosphodiesterase 6D, cGMP-specific, rod, delta	1,51	0,002
221830_at	RAP2A	RAP2A, member of RAS oncogene family	1,51	0,000
210694_s_at	MID1	midline 1 (Opitz/BBB syndrome)	1,51	0,000
200768_s_at	MAT2A	methionine adenosyltransferase II, alpha	1,51	0,002
213239_at	PIBF1	progesterone immunomodulatory binding factor 1	1,51	0,001
205285_s_at	FYB	FYN binding protein	1,51	0,001
203202_at	KRR1	KRR1, small subunit (SSU) processome component, homolog (yeast)	1,51	0,000
212332_at	RBL2	retinoblastoma-like 2 (p130)	1,51	0,000
220459_at	MCM3AP-AS1	MCM3AP antisense RNA 1	1,50	0,000
214850_at	SMA4	glucuronidase, beta pseudogene	1,50	0,001
205756_s_at	F8	coagulation factor VIII, procoagulant component	1,50	0,001
213853_at	DNAJC24	DnaJ (Hsp40) homolog, subfamily C, member 24	1,50	0,000
204256_at	ELOVL6	ELOVL fatty acid elongase 6	1,50	0,006
204048_s_at	PHACTR2	phosphatase and actin regulator 2	1,50	0,000
202318_s_at	SENP6	SUMO1/sentrin specific peptidase 6	1,50	0,000
210172_at	SF1	splicing factor 1	1,50	0,002
218095_s_at	TMEM165	transmembrane protein 165	1,50	0,001
221705_s_at	SIKE1	suppressor of IKBKE 1	1,50	0,000

Table S2. Probe sets in the *IGF1-R* signatures that are under-expressed in the high *IGF1-R* group

Probeset ID	Gene Symbol	Gene Title	Fold-Change (high vs. low)	P-value
242881_x_at	LOC100507460		-2,58	0,005
225685_at	CDC42EP3	CDC42 effector protein (Rho GTPase binding) 3	-1,88	0,000
225606_at	BCL2L11	BCL2-like 11 (apoptosis facilitator)	-1,82	0,004
226452_at	PDK1	pyruvate dehydrogenase kinase, isozyme 1	-1,79	0,001
235230_at	PHLDB2	pleckstrin homology-like domain, family B, member 2; phosphatidylinositol-specific phospholipase C, X domain containing 2	-1,77	0,003
225010_at	CCDC6	coiled-coil domain containing 6	-1,74	0,000
241819_at	TNFSF8	tumor necrosis factor (ligand) superfamily, member 8	-1,74	0,002
228116_at	FLJ39632	hypothetical LOC642477; hypothetical LOC400879	-1,73	0,003

226725_at	SLFN5	schlafen family member 5	-1,72	0,002
231990_at	USP15	ubiquitin specific peptidase 15	-1,71	0,000
227538_at	MED26	mediator complex subunit 26	-1,70	0,000
225044_at	NT5C3L	5'-nucleotidase, cytosolic III-like	-1,69	0,001
223085_at	RNF19A	ring finger protein 19A	-1,68	0,000
229256_at	PGM2L1	phosphoglucomutase 2-like 1	-1,68	0,002
226633_at	RAB8B	RAB8B, member RAS oncogene family	-1,67	0,001
236662_at	Hs.658292	Transcribed locus	-1,66	0,004
209392_at	ENPP2	ectonucleotide pyrophosphatase/phosphodiesterase 2	-1,63	0,004
227110_at	HNRNPC	heterogeneous nuclear ribonucleoprotein C (C1/C2)	-1,63	0,000
226158_at	KLHL24	kelch-like 24 (Drosophila)	-1,62	0,003
224164_at	TPM3	tropomyosin 3	-1,62	0,002
224836_at	TP53INP2	tumor protein p53 inducible nuclear protein 2	-1,62	0,001
226354_at	LACTB	lactamase, beta	-1,62	0,001
222853_at	FLRT3	fibronectin leucine rich transmembrane protein 3	-1,62	0,000
231882_at	FLJ39632		-1,61	0,001
241924_at	Hs.658154	Transcribed locus	-1,60	0,001
223584_s_at	KBTBD2	kelch repeat and BTB (POZ) domain containing 2	-1,60	0,000
237082_at	ASAP1	ArfGAP with SH3 domain, ankyrin repeat and PH domain 1	-1,60	0,001
225980_at	C14orf43	chromosome 14 open reading frame 43	-1,59	0,000
222700_at	ATL2	atlastin GTPase 2	-1,59	0,000
225582_at	ITPRIP	inositol 1,4,5-triphosphate receptor interacting protein	-1,59	0,003
230133_at	RC3H2	ring finger and CCCH-type zinc finger domains 2	-1,59	0,002
242999_at	Arhgef7	Rho guanine nucleotide exchange factor (GEF) 7	-1,58	0,004
225892_at	IREB2	iron-responsive element binding protein 2	-1,58	0,004
244137_at	KIAA0317	KIAA0317	-1,58	0,000
225154_at	SYAP1	synapse associated protein 1, SAP47 homolog (Drosophila)	-1,57	0,001
223336_s_at	RAB18	RAB18, member RAS oncogene family	-1,57	0,000
225519_at	PPP4R2	protein phosphatase 4, regulatory subunit 2	-1,57	0,000
228176_at	S1PR3	sphingosine-1-phosphate receptor 3	-1,56	0,000
222662_at	PPP1R3B	protein phosphatase 1, regulatory (inhibitor) subunit 3B	-1,56	0,000
226100_at	MLL5	myeloid/lymphoid or mixed-lineage leukemia 5 (trithorax homolog, Drosophila)	-1,56	0,000
230134_s_at	RC3H2	ring finger and CCCH-type zinc finger domains 2	-1,56	0,002
225564_at	SPATA13	spermatogenesis associated 13	-1,55	0,000
228536_at	PRMT10	protein arginine methyltransferase 10 (putative)	-1,54	0,000
228846_at	MXD1	MAX dimerization protein 1	-1,54	0,006
230082_at	LOC100133660	hypothetical LOC100133660	-1,54	0,005
227991_x_at	ZBTB43	zinc finger and BTB domain containing 43	-1,54	0,001
226155_at	Fam160b1	family with sequence similarity 160, member B1	-1,54	0,001
226394_at	MARCH05	membrane-associated ring finger (C3HC4) 5	-1,54	0,000
222900_at	NRIP3	nuclear receptor interacting protein 3	-1,54	0,001
225426_at	PPP6C	protein phosphatase 6, catalytic subunit	-1,54	0,000
224787_s_at	RAB18	RAB18, member RAS oncogene family	-1,54	0,000

226214_at	GDE1	glycerophosphodiester phosphodiesterase 1	-1,53	0,000
243601_at	LOC285957	hypothetical protein LOC285957	-1,53	0,000
242868_at	EPAS1	endothelial PAS domain protein 1	-1,53	0,001
226669_at	USP42	ubiquitin specific peptidase 42	-1,53	0,000
240674_at	JARID2	jumonji, AT rich interactive domain 2	-1,53	0,004
235407_at	Hs.662268	Transcribed locus	-1,53	0,000
223982_s_at	PNPLA8	patatin-like phospholipase domain containing 8	-1,53	0,002
235199_at	RNF125	ring finger protein 125	-1,52	0,006
243927_x_at	KIAA1429	KIAA1429	-1,52	0,004
223331_s_at	DDX20	DEAD (Asp-Glu-Ala-Asp) box polypeptide 20	-1,52	0,000
227160_s_at	C20orf7	chromosome 20 open reading frame 7	-1,52	0,001
227932_at	ARIH2	ariadne homolog 2 (Drosophila)	-1,52	0,000
224628_at	ERLEC1	chromosome 2 open reading frame 30	-1,51	0,000
226046_at	MAPK8	mitogen-activated protein kinase 8	-1,51	0,000
231182_at	WIPF1	WAS/WASL interacting protein family, member 1	-1,51	0,001
224705_s_at	TNRC6A	trinucleotide repeat containing 6A	-1,51	0,000
235699_at	REM2	RAS (RAD and GEM)-like GTP binding 2	-1,50	0,000
